# Postoperative Oral Dexamethasone and Its Effects on Pain, Trismus, and Quality of Life After Impacted Mandibular Third Molar Surgery: A Randomized Triple-Blind Split-Mouth Clinical Trial

**DOI:** 10.4317/jced.64067

**Published:** 2026-05-29

**Authors:** Thais Pimentel, Bruno Teixeira Gonçalves Rodrigues, Iolanda Zanotelli Lemos, Hugo Leonardo Mendes de Barros, Ramiro Beato Souza, Paulo José d’Albuquerque Medeiros, Danilo Passeado Branco Ribeiro

**Affiliations:** 1 DDS, OMFS, PhD. Oral &amp; Maxillofacial Surgery, Pedro Ernesto University Hospital, Rio de Janeiro State University, Rio de Janeiro, Brazil; 2 DDS, OMFS, MsC student. Oral &amp; Maxillofacial Surgery, Pedro Ernesto University Hospital, Rio de Janeiro State University, Rio de Janeiro, Brazil; 3 DDS, OMFS, MsC. Oral &amp; Maxillofacial Surgery, Pedro Ernesto University Hospital, Rio de Janeiro State University, Rio de Janeiro, Brazil; 4 DDS, OMFS, MsC. Oral &amp; Maxillofacial Surgery, São Marcos Hospital, Piauí, Brazil; 5 DDS, OMFS, PhD. Oral &amp; Maxillofacial Surgery, Pedro Ernesto University Hospital, Rio de Janeiro State University, Rio de Janeiro, Brazil

## Abstract

**Background:**

Surgical extraction of impacted third molars is associated with postoperative inflammatory complications such as pain and trismus. Corticosteroids are frequently used to reduce these sequelae. Therefore, the aim of this study was to evaluate the effects of postoperative oral dexamethasone on pain, trismus, and quality of life after surgical extraction of impacted mandibular third molars.

**Materials and Methods:**

A prospective, randomized, triple-blind, split-mouth clinical trial was conducted including 20 patients requiring bilateral extraction of impacted mandibular third molars. All participants received 8 mg of oral dexamethasone one hour before surgery. In the control protocol, patients received placebo tablets twice daily for three postoperative days, while in the test protocol patients received 4 mg of oral dexamethasone twice daily for three days. Pain intensity was recorded using a VAS at 24 hours, 72 hours, and 7 days after surgery. Maximum mouth opening (MMO) was measured preoperatively and on postoperative days 3 and 7. Quality of life was evaluated on day 7 using the Oral Health Impact Profile (OHIP-14). Statistical analyses were performed using paired tests with a significance level of 5%.

**Results:**

A total of 40 surgical procedures were performed. Mean surgical time did not differ significantly between groups. Pain scores were slightly lower in the test group during the early postoperative period, but no statistically significant differences were observed at any time point (p&gt;0.05). Both groups showed a reduction in MMO during the early postoperative period with partial recovery by day 7, without significant differences between treatments (p&gt;0.05). Quality-of-life assessment revealed no significant differences for most OHIP-14 domains, although the control group reported greater difficulty performing daily activities (p&lt;0.05).

**Conclusions:**

Postoperative administration of oral dexamethasone did not provide additional benefits in reducing pain, trismus, or most quality-of-life parameters compared with preoperative dexamethasone alone.

## Introduction

Extraction of impacted mandibular third molars is one of the most frequently performed procedures in oral and maxillofacial surgery (1). Postoperative sequelae such as pain, edema, and trismus are common consequences of surgical trauma and may negatively affect patients' quality of life during the early postoperative period (1 - 3). Regardless of the tooth position, depth, or angulation, surgical removal of impacted third molars may temporarily impair daily activities, including eating, speaking, and social interactions (1). Inflammation is a physiological response of the immune system triggered by tissue injury. The classical signs of inflammation include hyperalgesia, edema, leukocyte recruitment, changes in local blood flow and increased vascular permeability. During the inflammatory process, damaged tissues release several chemical mediators such as prostaglandins, histamine and other inflammatory cytokines that can lower the pain threshold of peripheral sensory nerve fibers (1 - 3). Consequently, several pharmacological protocols have been proposed to reduce the intensity of postoperative inflammatory symptoms associated with third molar surgery (3 - 5). Corticosteroids are widely used in oral surgery due to their potent anti-inflammatory properties. These drugs act by inhibiting phospholipase A2 activity, thereby reducing the synthesis of arachidonic acid derivatives and suppressing the release of inflammatory mediators such as prostaglandins and leukotrienes. In addition, corticosteroids decrease capillary permeability, leukocyte migration and neutrophil accumulation at the site of inflammation, ultimately reducing postoperative pain, swelling, and trismus (3 - 5). Among corticosteroids, dexamethasone is one of the most commonly used agents in oral and maxillofacial surgery. It is a synthetic glucocorticoid derived from prednisolone, characterized by a long biological half-life and approximately 20-30 times greater anti-inflammatory potency than hydrocortisone, with minimal mineralocorticoid activity (3 , 4) Dexamethasone has been investigated through different routes of administration, including oral, intravenous, intramuscular, and submucosal administration, during both preoperative and postoperative periods (5 - 7). Among corticosteroids, dexamethasone is one of the most commonly used agents in oral and maxillofacial surgery. It is a synthetic glucocorticoid derived from prednisolone, characterized by a long biological half-life and greater anti-inflammatory potency, with minimal mineralocorticoid activity (3 , 4). Dexamethasone has been investigated through different routes of administration, including oral, intravenous, intramuscular, and submucosal routes, in both preoperative and postoperative periods (5 - 7). A systematic review conducted by Almeida et al. (5) evaluated randomized clinical trials comparing corticosteroids with placebo after third molar surgery and demonstrated significant benefits in reducing postoperative pain, swelling, and trismus. However, concerns remain regarding potential adverse effects associated with prolonged corticosteroid use, including adrenal suppression, increased susceptibility to infections, and delayed wound healing (6). Nevertheless, available evidence suggests that such complications are unlikely when corticosteroids are administered as a single preoperative dose or for short postoperative periods of less than five days (7 - 10). Therefore, the aim of this randomized, triple-blind, split-mouth clinical trial was to evaluate the effects of postoperative oral dexamethasone on pain, trismus, and quality of life in patients undergoing surgical extraction of impacted mandibular third molars.

## Materials and Methods

**Figure 1:**
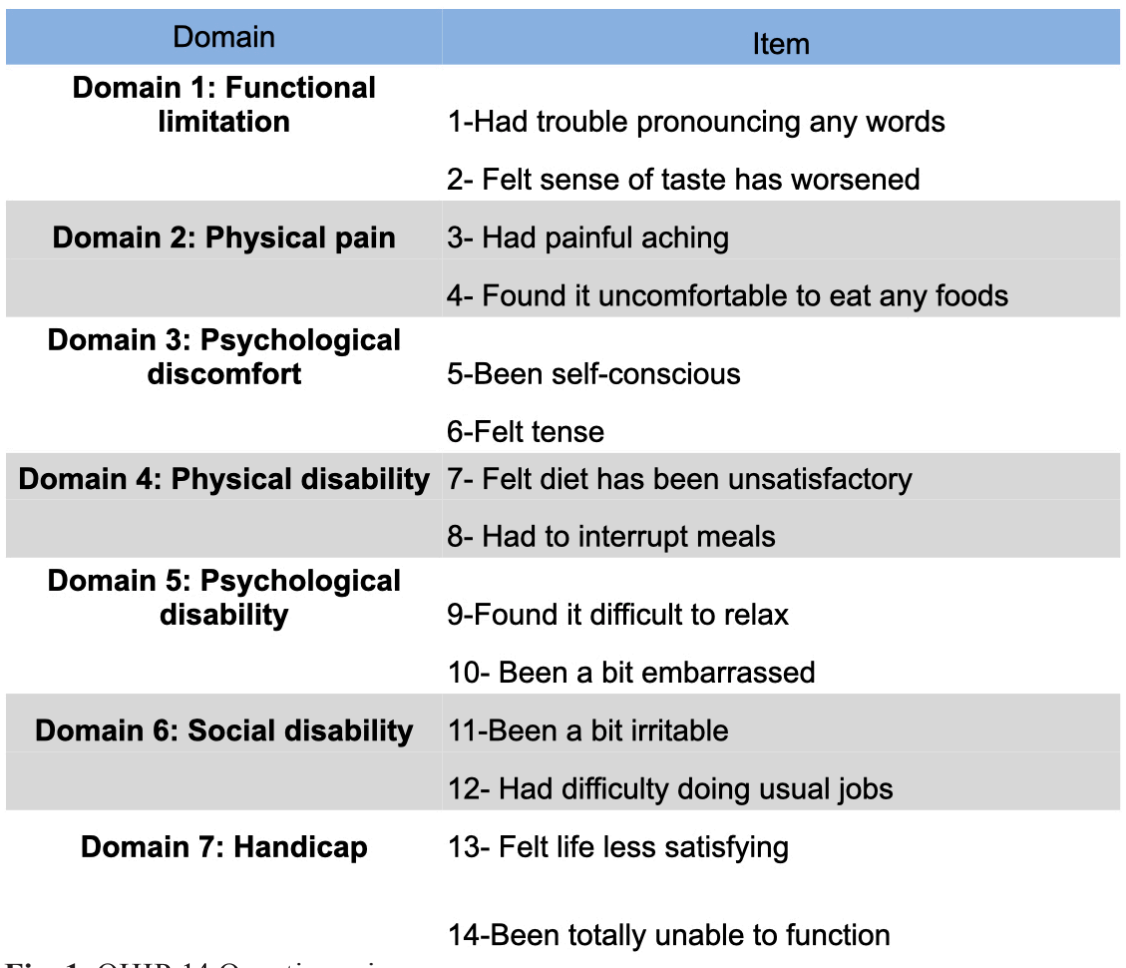
OHIP-14 Questionnaire.

This study was designed as a prospective, randomized, triple-blind, split-mouth clinical trial conducted between November 2022 and June 2023. Twenty patients requiring bilateral surgical extraction of impacted mandibular third molars were included. Each participant underwent two surgical procedures, with a minimum interval of 30 days between surgeries. The study protocol was approved by the institutional ethics committee of Pedro Ernesto University Hospital, Rio de Janeiro State University, Brazil (CAAE: 42338820.4.0000.5259), and conducted in accordance with the Declaration of Helsinki. The trial was prospectively registered in the Brazilian Registry of Clinical Trials (ReBEC) under the number RBR-4xjvsqh. All participants provided written informed consent prior to enrollment. Patients were recruited from individuals referred for surgical extraction of impacted mandibular third molars. The inclusion criteria were: age between 16 and 40 years, American Society of Anesthesiologists (ASA) physical status I, indication for bilateral mandibular third molar extraction, and similar impaction characteristics on both sides. The exclusion criteria included the presence of pericoronitis in the mandibular third molar region, history of allergy or hypersensitivity to any medication used in the study, and systemic conditions contraindicating the use of corticosteroids. Randomization was performed using a computer-generated random number table to determine which side would receive the test or control protocol. The allocation sequence was concealed by an independent investigator who was not involved in the surgical procedures or outcome assessment, and allocation information remained sealed until completion of data collection. In all participants, the right mandibular third molar was extracted during the first surgical appointment and the left molar during the second procedure. The study followed a triple-blind design, in which patients, the surgeon and the investigator responsible for outcome assessment were all blinded to group allocation. Dexamethasone and placebo tablets were identical in appearance, packaging, and administration schedule to ensure adequate blinding. Both groups received a preoperative dose of dexamethasone; one hour before surgery, patients received two 4 mg oral dexamethasone tablets (total dose of 8 mg). However, postoperative medication differed between groups: patients in the control group received placebo tablets twice daily for 3 days after surgery, whereas patients in the test group received 4 mg of oral dexamethasone twice daily for 3 days after surgery. All surgical procedures were performed by the same experienced surgeon using a standardized technique. A mucoperiosteal flap was elevated in an anteroposterior direction without extension beyond the external oblique ridge. Osteotomy was performed around the crown, followed by odontosection using surgical burs under copious irrigation with sterile saline solution. The tooth was sectioned and removed using a straight elevator. After extraction, the socket was irrigated with sterile saline solution, and primary closure was achieved using 3-0 silk sutures. All patients received the same postoperative pharmacological regimen consisting of amoxicillin (500 mg every 8 hours) and acetaminophen (750 mg every 6 hours) for five days. The evaluated outcomes were pain, trismus, and postoperative quality of life. Postoperative pain was assessed using a 10-cm visual analog scale (VAS), where 0 indicated "no pain" and 10 represented "the worst pain imaginable." Patients recorded pain intensity at 24 hours, 72 hours, and 7 days after surgery. Maximum mouth opening (MMO) was measured as the distance between the incisal edges of the upper and lower central incisors, with measurements obtained preoperatively and on postoperative days 3 and 7. Each measurement was performed three times, and the average value was used for statistical analysis. Postoperative quality of life was assessed using the Oral Health Impact Profile questionnaire (OHIP-14) (11), which consists of 14 items distributed across seven domains and evaluates the impact of oral health on daily activities. Each item was scored using a five-point Likert scale ranging from 0 (never) to 4 (always), and patients completed the questionnaire on the seventh postoperative day following each surgical procedure, (Fig. 1).

1

Data analysis was performed using appropriate statistical tests after assessment of data distribution. The Kolmogorov-Smirnov test was used to evaluate normality. For non-normally distributed variables, the Wilcoxon signed-rank test was applied, whereas for normally distributed variables, comparisons between groups were performed using paired Student's t-tests. A significance level of 5% ( = 0.05) was adopted for all statistical analyses.

## Results

**Figure 2:**
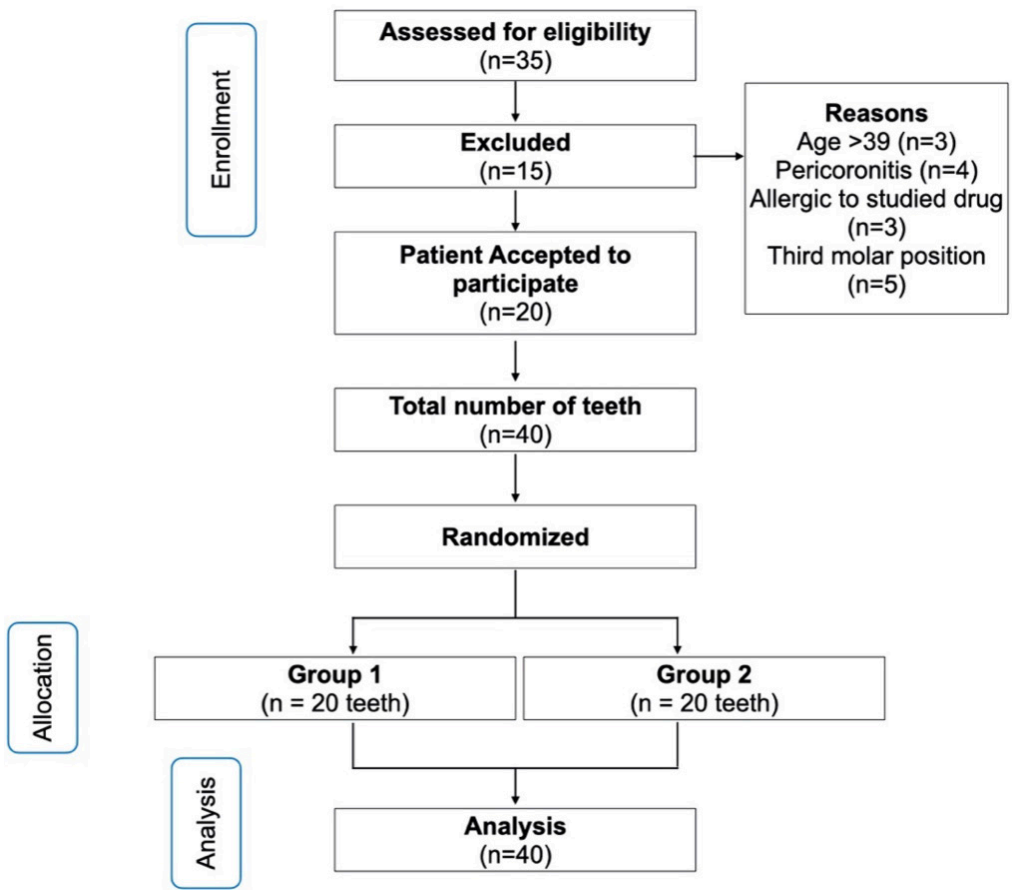
CONSORT flow diagram of participant recruitment, eligibility assessment, randomization, allocation, and analysis.

A total of 20 patients (13 females and 7 males) were included in this randomized split-mouth clinical trial, resulting in 40 surgical procedures. The participants' ages ranged from 16 to 37 years, with a mean age of 23 years (SD ± 3.6). All participants completed both surgical procedures and follow-up evaluations (Fig. 2).

2

**Figure 3:**
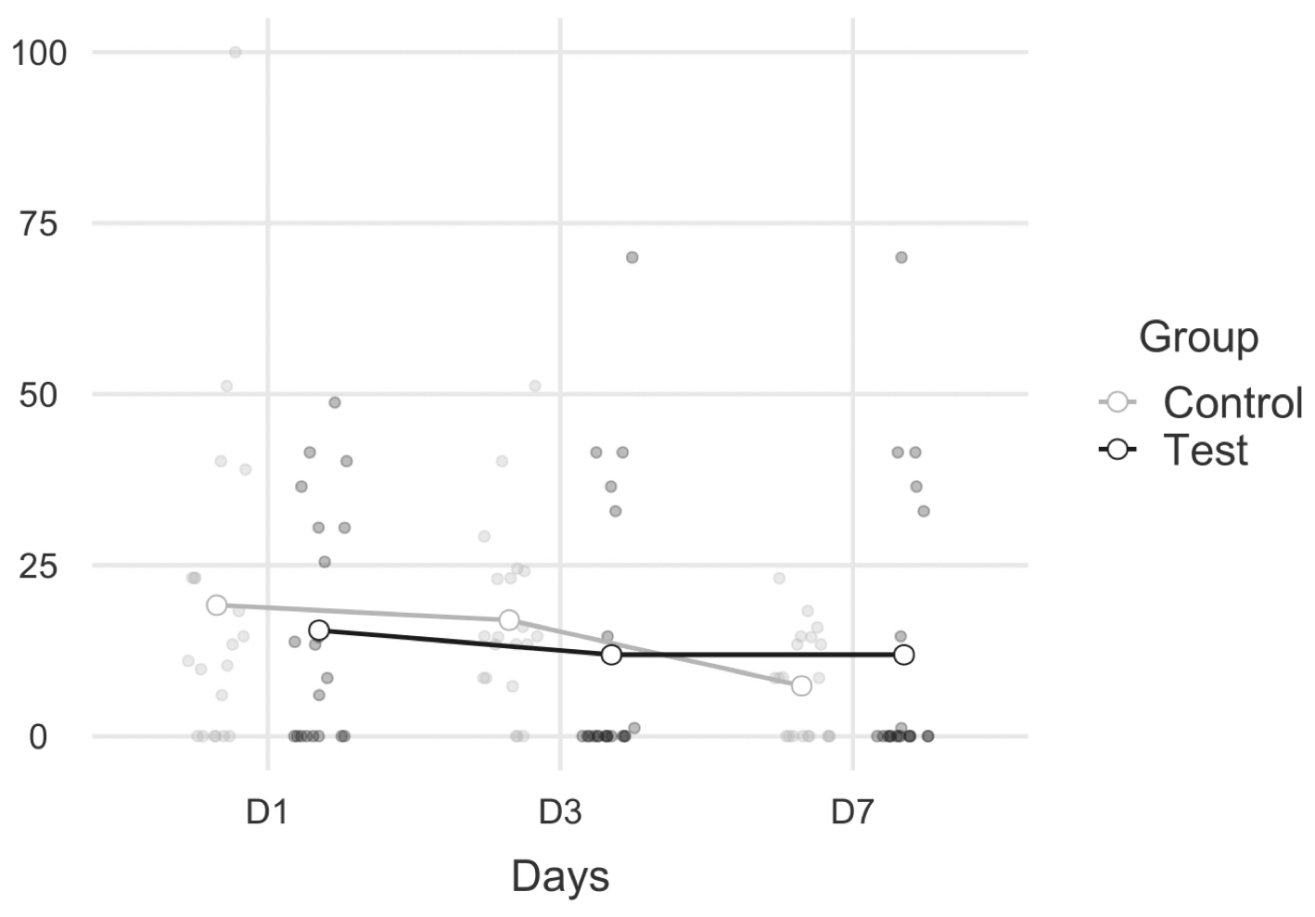
Postoperative pain scores measured using the visual analog scale (VAS) at 24 hours (D1), 72 hours (D3), and 7 days (D7) after mandibular third molar surgery. Individual data points represent pain scores reported by each patient, while the lines indicate mean values for the control group (preoperative dexamethasone + postoperative placebo) and the test group (preoperative dexamethasone + postoperative dexamethasone). No statistically significant differences were observed between groups at any evaluated time point (p &gt; 0.05).

The mean surgical time was 12.36 minutes (SD ± 5.70), ranging from 6.16 to 29 minutes. The average surgical time was 12.3 minutes (SD ± 5.7) for the control group and 12.9 minutes (SD ± 5.1) for the test group, with no statistically significant difference between groups (p &gt; 0.05). Pain score distribution was assessed using the Kolmogorov-Smirnov test, which indicated a non-normal distribution (p &lt; 0.05). Therefore, comparisons between groups were performed using the Wilcoxon signed-rank test. Mean pain scores at 24 hours (D1) were 19.16 (SD ± 24.1) in the control group and 15.48 (SD ± 16.9) in the test group (p &gt; 0.05). At 72 hours (D3), mean values were 16.9 (SD ± 12.9) for the control group and 11.9 (SD ± 20.6) for the test group (p &gt; 0.05). On postoperative day 7 (D7), the mean pain score was 7.3 (SD ± 7.6) in the control group and 11.9 (SD ± 20.6) in the test group (p &gt; 0.05). Although pain scores tended to be lower in the test group during the early postoperative period, no statistically significant differences were observed between groups at any evaluated time point (Tables 1,2, Fig. 3).

Table 1

Table 2

3

For trismus, normality and homogeneity of variance were confirmed using the Kolmogorov-Smirnov and Levene tests. Consequently, comparisons were performed using paired Student's t-tests. The mean maximum mouth opening (MMO) on postoperative day 1 was 47.35 mm (SD = 6.69) in the control group and 45.3 mm (SD = 7.99) in the test group (p &gt; 0.05). On day 3, the mean MMO was 40.4 mm (SD = 9.81) in the control group and 39.15 mm (SD = 8.94) in the test group (p &gt; 0.05). On day 7, the mean values were 43.9 mm (SD = 7.3) and 42.55 mm (SD = 9.77) for the control and test groups, respectively (p &gt; 0.05). These findings indicate that both groups experienced a reduction in mouth opening during the early postoperative period, followed by partial recovery by day 7, with no statistically significant differences between treatments (Tables 3,4).

Table 3

Table 4

**Figure 4:**
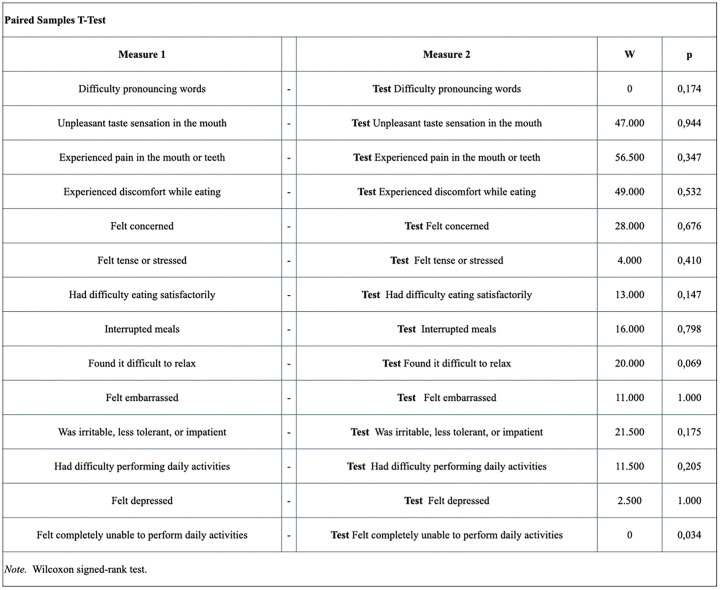
P-values obtained from paired samples t-tests comparing the control and test groups for each item of the OHIP-14 questionnaire. No statistically significant differences were observed between groups, but for the item “Have you felt totally unable to perform your daily activities?”, with a higher impact reported in the control group (p 0.05).

Postoperative quality of life was assessed using the Oral Health Impact Profile (OHIP-14) questionnaire on the seventh postoperative day, and comparisons between groups were performed using the Wilcoxon signed-rank test. No statistically significant differences were observed between the control and test groups for most OHIP-14 items (p &gt; 0.05). However, a statistically significant difference was identified for the item "Have you felt totally unable to perform your daily activities?", with a higher impact reported in the control group (p &lt; 0.05) (Fig. 4).

4

## Discussion

Despite the available evidence indicating that the main clinical benefits of dexamethasone are achieved with preoperative administration (4 - 9), corticosteroids continue to be prescribed using a wide range of protocols in oral surgery (6 - 8). Considerable variability persists regarding dosage, timing, route of administration, and duration of therapy, including the continued use of postoperative regimens (4 - 11). This heterogeneity likely reflects the absence of universally accepted clinical guidelines and the inconsistency of findings across studies. As a result, many clinicians maintain empirical prescribing practices based on personal preference or training rather than strictly evidence-based protocols. In this context, the present study contributes to the existing literature by specifically evaluating the additional effect of postoperative oral dexamethasone, helping to clarify whether extending corticosteroid therapy beyond the preoperative period is clinically justified. In the present study, all patients received a preoperative dose of dexamethasone, while only the test group received additional postoperative dexamethasone for three days. The results demonstrated that postoperative administration did not result in statistically significant improvements in pain, trismus, or most quality-of-life parameters when compared with preoperative administration alone. Although pain scores tended to be slightly lower in the test group during the early postoperative period, these differences did not reach statistical significance. These findings suggest that the anti-inflammatory effect achieved by a single preoperative dose of dexamethasone may be sufficient to control most inflammatory symptoms following routine third molar surgery. Therefore, extending corticosteroid therapy into the postoperative period may not provide additional clinical benefit in healthy patients undergoing uncomplicated procedures. The results of the present study are consistent with previous investigations that have reported limited additional benefits associated with prolonged corticosteroid administration (12 - 20). Variability among clinical trials may be explained by differences in drug dosage, route of administration, timing of drug delivery, and surgical complexity. Furthermore, surgical factors such as operative time, extent of bone removal, and surgeon experience may influence postoperative outcomes and potentially mask pharmacological effects. The route of administration may also play an important role in the clinical effectiveness of corticosteroids (16). While submucosal and intramuscular administration have been proposed to increase local drug concentration, oral administration remains a practical and convenient alternative in outpatient settings. Oral dexamethasone is rapidly absorbed from the gastrointestinal tract and reaches effective systemic concentrations within a relatively short period, supporting its use in perioperative management, as observed in this trial (15 - 18). In the present study, trismus was observed in both groups during the early postoperative period, followed by partial recovery by the seventh postoperative day. However, no significant differences were detected between groups. This finding is consistent with previous studies suggesting that corticosteroids do not directly affect muscle function but may reduce trismus indirectly by attenuating the inflammatory response associated with surgical trauma (6 , 15). Postoperative quality of life was assessed using the OHIP-14 questionnaire on the seventh postoperative day. No significant differences were observed between groups for most domains. This may be related to the timing of the evaluation, as postoperative symptoms tend to improve substantially within the first week. Earlier assessments, particularly within the first 48 to 72 hours after surgery, might have been more sensitive in detecting differences between treatment protocols. Some limitations of this study should be acknowledged. First, the relatively small sample size may have limited the statistical power to detect subtle differences between groups. Second, the surgical sequence was not randomized, as the right side was consistently operated on first, which may have introduced a potential order-related bias. Additionally, individual variability in inflammatory response and pain perception may have influenced postoperative outcomes despite the split-mouth design. Furthermore, postoperative edema was not included as an outcome measure. Although swelling is a clinically relevant parameter in third molar surgery, its assessment often relies on subjective or indirect measurement methods, which may compromise accuracy and reproducibility. In the present study, priority was given to outcomes with greater objectivity and clinical applicability, such as pain, maximum mouth opening, and patient-reported quality of life. Despite these limitations, the randomized triple-blind split-mouth design represents a significant methodological strength, as it minimizes interindividual variability and reduces potential sources of bias. In addition, the use of a standardized surgical technique and a single experienced surgeon contributed to improved internal validity.

## Conclusions

Postoperative administration of oral dexamethasone did not provide additional benefits in reducing pain, trismus, or most quality-of-life parameters when compared with preoperative dexamethasone alone following impacted mandibular third molar surgery. These findings reinforce current evidence that a single preoperative dose may be sufficient to control postoperative inflammatory symptoms, questioning the routine extension of corticosteroid therapy into the postoperative period. Furthermore, future studies should also investigate different dosages, routes of administration, and timing protocols, as well as include comprehensive assessment of postoperative morbidity, to better define optimized anti-inflammatory strategies for third molar surgery.

## Figures and Tables

**Table 1:** Paired comparisons of postoperative pain scores between control and test groups at different time points (D1, D3, and D7). Statistical analysis was performed using the Wilcoxon signed-rank test due to non-normal data distribution. Data are presented as W values and corresponding p-values. No statistically significant differences were observed between groups at any evaluated time point (p > 0.05).

Control		Test	W	p
Pain D1 Control	-	Pain D1 Test	76.000	0,698
Pain D3 Control	-	Pain D3 Test	124.000	0,098
Pain D7 Control	-	Pain D7 Test	54.000	0,755

1

**Table 2:** Postoperative pain scores at different time points (D1, D3, and D7) for control and test groups. Data are presented as mean and standard deviation (SD). Pain intensity was assessed using a visual analog scale (VAS). Statistical comparisons between groups were performed using the Wilcoxon signed-rank test due to non-normal data distribution. No statistically significant differences were observed between groups at any evaluated time point (p > 0.05).

	Group	N	Mean	SD
Pain D1	Control	20	19.166	24.180
	Test	20	15.483	16.942
Pain D3	Control	20	16.977	12.978
	Test	20	11.910	20.694
Pain D7	Control	20	7.365	7.642
	Test	20	11.910	20.694

2

**Table 3:** Comparison of maximum mouth opening (mm) between control and test groups at different postoperative time points (D1, D3, and D7). Data are presented as mean and standard deviation (SD). Statistical comparisons between groups were performed using the paired Student’s t-test, considering the split-mouth design of the study. No statistically significant differences were observed between groups at any evaluated time point (p > 0.05).

	Group	N	Mean	SD
Trimus D1	Control	20	47.350	6.691
	Test	20	45.300	7.994
Trimus D3	Control	20	40.400	9.816
	Test	20	39.150	8.946
Trimus D7	Control	20	43.950	7.330
	Test	20	42.550	9.779

3

**Table 4:** Paired comparisons of maximum mouth opening (mm) between control and test groups at different postoperative time points (D1, D3, and D7). Statistical analysis was performed using the paired Student’s t-test. Data are expressed as t-values, degrees of freedom (df), and corresponding p-values. No statistically significant differences were observed between groups at any evaluated time point (p > 0.05).

		Test	t	df	p
Trimus D1 control	-	Trimus D1 test	1.853	19	0,079
Trimus D3 control	-	Trimus D3 test	0,478	19	0,638
Trimus D7 control	-	Trimus D7 test	0,580	19	0,569

4

## Data Availability

The datasets used and/or analyzed during the current study are available from the corresponding author.
